# Bridging knowledge gaps for healthy China 2030 plan: a province-wide assessment of primary healthcare workers' competencies and training preferences in Henan, China

**DOI:** 10.3389/fpubh.2026.1762052

**Published:** 2026-02-26

**Authors:** Clifford Silver Tarimo, Yifei Feng, Quanman Li, Yuefeng Bi, Yao Song

**Affiliations:** 1 School of Pharmaceutical Science, Zhengzhou University, Zhengzhou, Henan, China; 2 Department of Health Management, College of Public Health, Zhengzhou University, Zhengzhou, Henan, China; 3 Department of Science and Laboratory Technology, Dar es Salaam Institute of Technology, Dar es Salaam, Tanzania; 4 School of Computer Science and Artificial Intelligence Zhengzhou University, Zhengzhou, Henan, China

**Keywords:** China, Henan, knowledge gaps, primary healthcare workers, training preferences

## Abstract

**Background:**

Primary healthcare workers (PHCWs) are foundational to equitable health service delivery. In 2016, China launched the Healthy China 2030 plan, the first medium- to long-term national health strategy which elevates disease prevention, health promotion and universal coverage as national priorities. Addressing knowledge gaps in the PHC workforce is therefore essential to achieving the plan's goal of raising average life expectancy to 79 years by 2030. We examined PHCWs' self-perceived knowledge gaps and training preferences across Henan province to understand how workforce development can support Healthy China 2030.

**Methods:**

A cross-sectional survey of 119,051 PHCWs across Henan was conducted in August 2023. Participants were recruited from village health clinics, township health centers, community health service centers, and health management institutions. Respondents included healthcare workers across seniority levels, ranging from junior level, intermediate level, associate senior level, to senior level. Data on self-perceived knowledge gaps (basic theory, professional knowledge, and practical skills) and training preferences (Theory-based, Case-sharing, Interactive discussion, and Skills simulation) were analyzed using descriptive statistics, Generalized Linear Models (GLM), and K-Means clustering.

**Results:**

Significant knowledge deficiencies were reported by 40.0% of PHCWs across all the tested domains, with notable gaps in practical skills (15.7%) and professional knowledge (14.1%). Gaps were most pronounced in rural-based clinics and among junior staff. PHCWs strongly preferred experiential learning like case sharing (76.4%) and skills simulation (75.2%). Clustering analysis identified six distinct knowledge gaps and training preference profiles.

**Conclusion:**

Henan's PHC workforce faces pervasive knowledge gaps that threaten progress toward the prevention-oriented, equitable healthcare envisioned by Healthy China 2030. Policymakers should design competency-based training programs that prioritize rural clinics, integrate digital and hands-on modalities, and reinforce health promotion and chronic disease management. Establishing regional training hubs, mentorship systems, and rigorous evaluation mechanisms will help build a workforce capable of advancing China's national goals of improving health literacy, reducing chronic disease burdens, and achieving universal health coverage.

## Introduction

Primary healthcare (PHC) is the cornerstone of any health system, serving as the first point of contact for individuals seeking medical care. As part of China's healthcare reform strategy to achieve universal and fair access to high-quality medical services, significant emphasis has been placed on strengthening PHC (1, 2). This national commitment was formalized in the Healthy China 2030 Plan, which establishes disease prevention, health promotion, and equitable primary healthcare as central pillars of China's long-term health strategy. Healthy China 2030 Plan emphasizes strengthening the PHC workforce, improving service capabilities, and advancing the shift from treatment-oriented to prevention-centered care (3, 4). Achieving these goals depends fundamentally on developing a competent PHC workforce capable of delivering standardized, community-focused, and people-centered services across diverse regions. China's PHC system operates through a tiered network of facilities that serve as the foundational units of healthcare delivery (5). These facilities may include Community Health Service Centers (CHSCs), Health Management Institutions, Township Health Centers (THCs), and Village Health Clinics (VHCs), each serving distinct but complementary roles. CHSCs, established primarily in urban areas, are responsible for providing a wide range of services including medical care, public health, disease prevention, health education, and rehabilitation (6). Health management institutions specialize mostly in preventive care and health promotion including screening for non-communicable diseases, health education campaigns, and maternal and child health services (7). Township Health Centers (THC) are the cornerstone of rural health services. Located at the township level, THCs typically have inpatient capacity and are usually staffed with general practitioners, nurses, and sometimes traditional Chinese medicine (TCM) practitioners (8). Village Health Clinics represent the most grassroots-level facilities in China's PHC system. Operated often by “village doctors” (previously known as “barefoot doctors”), VHCs provide essential care such as treatment for common illnesses, blood pressure monitoring, and referral to higher-level facilities and are only source of care for village residents (9). Across these facility types, primary healthcare workers typically receive a combination of pre-service academic training and in-service continuing education. Continuing education is commonly delivered through institution-based training sessions, regional workshops organized by health authorities, short-term clinical rotations, and increasingly through digital learning platforms and online courses, particularly for providers in rural and underserved settings (10, 11). However, the scope, frequency, and practical orientation of these training opportunities can vary substantially across facility levels and geographic contexts. PHC services plays an important role in delivering preventive, curative, and rehabilitative services, particularly in rural and underserved communities. The importance of PHC is further emphasized in the Healthy China 2030 Plan, which outlines strategic priorities for health system development with a strong focus on primary care (12–14). Despite significant financial investments and extensive infrastructure development over the past decade, evidence indicates that the quality of PHC in China remains subpar with persistent inequities in service quality (2). This shortfall has led to missed opportunities for enhancing population health and service delivery (15, 16). The effectiveness of PHC hinges on the competency and preparedness of healthcare professionals, often influenced by regional disparities in training, resources, and infrastructure. While prior studies have extensively documented gaps in infrastructure and financing, limited attention has been paid to the competency gaps among PHC workers and their alignment with evolving clinical and community needs. Current evidence suggests that regional disparities in training quality, outdated pedagogical approaches, and insufficient continuing education opportunities hinder the ability of PHC providers to deliver patient-centered, prevention-focused care (17, 18). The rapid integration of digital health technologies and shifting patient expectations demand new skill sets that traditional training programs may not adequately address (19). When the knowledge gaps on essential skills remain prevalent, patients are prone to receiving suboptimal care. This may include misdiagnosis, improper disease management, or delayed referrals (20). Such gaps are common in under-resourced areas despite recent improvements in China's healthcare system whereby rural regions have been left with severely limited medical resources and training opportunities (21). In practice, this means a village doctor might struggle to manage a stroke or any routine cases due to insufficient training or nurses at a township clinic may not be familiar with the latest hypertension guidelines. Consequently, many patients avoid local clinics altogether, turning instead to overburdened regional or provincial hospitals even for minor health concerns. This pattern not only overwhelms higher-level facilities but also reflects a broader distrust in community-based healthcare services. Beyond clinical knowledge, research has also reported that barriers in communication and knowledge-sharing among PHC workers may be playing a role. Studies highlight that limited trust and unfamiliarity among colleagues hinder effective teamwork, ultimately impeding effective communication in patient care (22). From a conceptual perspective, workforce “knowledge gaps” in primary healthcare can be understood as existing discrepancies between the competencies required to deliver standardized, prevention-oriented, and patient-centered care, and the competencies that healthcare workers perceive they currently possess. Such gaps are not merely individual deficits, but systemic signals of misalignment between evolving service demands, training provision, and practice environments. Training Needs Analysis (TNA) frameworks offer a theoretically grounded approach for identifying such discrepancies and prioritizing capacity-building interventions. Evidence from international primary care and integrated-care reforms demonstrates that theory-informed assessments of workforce competencies are essential for designing effective, context-appropriate, and sustainable training strategies, particularly in health systems characterized by workforce heterogeneity and increasing demands for continuity and coordination of care (23–25). Addressing these persistent competency gaps requires a structured and validated approach to assessing training needs. Within this theoretical tradition, the Hennessy–Hicks Training Needs Analysis (TNA) Framework (26), endorsed by the World Health Organization (WHO), provides a systematic method for identifying knowledge deficits, practical skill gaps, and priority areas for workforce development within healthcare teams. The framework has been widely used in diverse health systems to guide capacity-building initiatives, ensuring that training efforts align with actual performance requirements. Guided by this framework, the present study assesses PHC workers' self-perceived knowledge gaps and training preferences to generate evidence that can strengthen PHC workforce competencies in line with Healthy China 2030 priorities. China has implemented a various training program to strengthen its healthcare workforce, from modern digital platforms to traditional hands-on apprenticeships, targeting various levels of healthcare providers from village doctors to medical residents. Digital learning has rapidly become a cornerstone of continuing education for Chinese healthcare workers. Online platforms and mobile applications allow healthcare providers, especially those in marginalized areas, to get access to up-to-date medical knowledge without leaving their posts. For example, the China-Gates Foundation Tuberculosis Control Program established a national telemedicine and training platform with live-streamed lectures and on-demand modules for TB care (27). This blended synchronous/asynchronous e-learning model enabled county-level clinicians and primary care staff to learn advanced TB management at their own pace. Mobile technology has further enhanced digital learning. WeChat, China's ubiquitous messaging mobile application, has been repurposed as an educational tool in healthcare. In one county, a WeChat-based training program was used to deliver weekly content on tuberculosis management to village doctors for 1 year (28). Policy support has also been crucial as the National Health Commission (NHC) and other authorities often endorse and fund online training, lending credibility, and ensuring these programs align with national health priorities (10). While digital education is warranted, hands-on experience remains irreplaceable in building clinical skills. China has a long tradition of workshop-style trainings and practical rotations to improve healthcare workers' competencies. A prominent example is the grassroots medical workers training program that was launched by the NHC in 2018, which by 2023 had enrolled already 650,000 providers. Ninety-six accredited medical institutions were designated as training bases, where trainees can learn on-site in well-equipped settings under expert supervision (29). Mentorship programs in healthcare have been shown to successfully pair less-experienced workers with seasoned mentors, facilitating knowledge transfer, and professional support. Additionally, mentorship programs in China have proven particularly beneficial in bridging the transition from training to independent practice. A compelling case study from a tertiary hospital in Zhejiang highlights the effectiveness of one-on-one mentorship initiative for newly graduated nurses whereby new recruit was assigned an experienced nurse mentor during their initial year of employment, yielding remarkable results. The turnover rate among mentored nurses plummeted to just under 4% in their first year, compared to 14% in a control group that received standard orientation training (30). The current study was conducted in 2023, during a period when COVID-19 control measures in China were easing and primary healthcare systems were transitioning from emergency response toward routine service delivery. This phase followed rapid shifts in service delivery models, workforce responsibilities, and the expanded use of digital training and learning platforms within primary care. As a result, the patterns of perceived competency gaps and training preferences observed in this study reflect a health system in transition, shaped by evolving post-pandemic service demands and workforce expectations. While previous studies have identified knowledge gaps among PHC workers, most lack granular insights into regional variations in training needs or preferences, particularly in provinces facing compounded demographic challenges. Henan province, home to nearly 100 million (31) residents and characterized by stark urban-rural disparities (32), aging populations (33), and high burdens of chronic disease (34), serves as a critical microcosm of these challenges. With over 60% of its population residing in rural areas and an overstretched PHC workforce (35), Henan's struggles mirror systemic issues faced by many central and western Chinese provinces, yet remain under-represented in literature. The current research transcends the limitations of smaller, siloed studies to provide a comprehensive, province-wide assessment of knowledge gaps and training preferences, enabling policymakers to tailor interventions to Henan's unique epidemiological and socio-economic landscape while offering transferable lessons for provinces with similar PHC challenges.

## Methodology

### Study design, data collection, setting, and study population

This cross-sectional survey analyzed self-reported knowledge gaps and training preferences of 119,051 primary healthcare workers (PHCWs) across 17 prefectures in Henan Province, China (population ~100 million), a region with significant urban-rural healthcare disparities. Data were collected in August 2023. The target population included PHCWs from diverse facilities (village/township/community health centers, health management institutions). A comprehensive coverage approach using an electronically distributed, pilot-tested questionnaire maximized response volume across varied contexts. The survey was disseminated through official administrative channels coordinated by local health authorities at the provincial and prefectural levels. Facility managers and designated coordinators facilitated distribution of the electronic questionnaire to eligible primary healthcare workers within their institutions. Participation was voluntary, and respondents completed the survey anonymously using a secure online platform, which helped ensure broad geographic coverage across all facility types and professional levels.

### Knowledge disparities, training preferences, and adopted frameworks

Knowledge gaps were evaluated by asking participants to identify areas they believe require improvement to enhance their service capabilities. Respondents were provided with multiple-choice options, including insufficient understanding of basic theory, limited professional knowledge, and inadequate practical skills. China's PHC practitioners have diverse training backgrounds, ranging from 3 year vocational diplomas to 8 year medical degrees (2). This educational diversity creates varying levels of foundational knowledge, making it critical to identify basic theory gaps as a first step in developing targeted training interventions. The assessment of professional knowledge gaps was inspired by China's ongoing healthcare reform initiatives, particularly the transition from a disease-specific to a comprehensive primary care model. The Healthy China 2030 initiative explicitly calls for strengthening professional knowledge among PHC workers to address the changing disease burden landscape (3). Additionally, China's rapid medical advancement and the continuous evolution of clinical guidelines necessitate regular assessment of professional knowledge gaps. The “know-do gap” has been identified as a key barrier to high-quality healthcare in China (36), making the assessment of professional knowledge deficiencies essential for improving care quality. The assessment of practical skills gaps was influenced by the documented disconnect between theoretical training and clinical practice in China's medical education system. Studies have shown that many PHC workers in China possess theoretical knowledge but lack the practical skills to implement this knowledge effectively (37). China's healthcare system is rapidly adopting new technologies and treatment modalities, creating a need for continuous practical skills development, hence making the assessment of practical skills gaps.

### Conceptual framework

**Figure 1:**
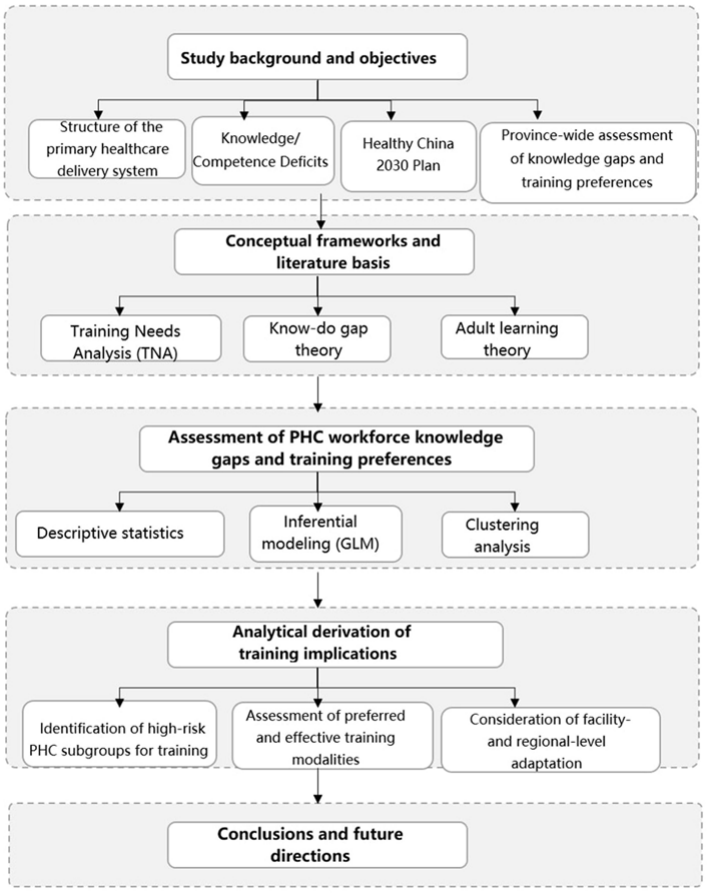
Conceptual framework and analytical phases of the study.

Our questionnaire (Supplementary file 1) design was informed by the Hennessy-Hicks Training Needs Analysis Framework, a validated methodology endorsed by the World Health Organization (WHO) for identifying healthcare worker training needs (26). This framework employs a gap analysis approach that compares the importance of specific tasks with current performance levels to identify priority areas for training interventions. While our questionnaire was not a direct application of the Hennessy-Hicks instrument, it follows the same methodological principles of assessing knowledge gaps and training preferences to inform targeted professional development interventions. The question on knowledge gaps aligns with the Hennessy-Hicks framework's approach to identifying areas where performance improvement is needed. While the original framework asks respondents to rate both criticality and performance for each task, our questionnaire directly asked healthcare workers to identify areas where they perceived gaps in their knowledge or skills, effectively capturing the “gap” component of the framework. We analyzed the frequency of reported knowledge gaps across different domains and also quantified their training preferences. Figure 1 illustrates the conceptual framework, analytical workflow, and derivation of training implications that guided the study.

### Statistical analyses

#### Descriptive statistics

Descriptive statistics were calculated for demographic variables and knowledge gap responses, with categorical data summarized as frequencies/percentages (95% CIs via Wilson method). Knowledge gaps (insufficient basic theory, professional knowledge, practical skills) and training preferences (theory-based, case sharing, skills simulations, interactive discussions) were analyzed singularly and in combination. Chi-square tests (*p* < 0.05) examined associations between participant characteristics (gender, facility type, seniority, region) and reported gaps/preferences. To visualize geographic disparities, city-level survey responses were linked with Henan Province administrative boundaries; self-reported gaps were aggregated by city and displayed on a choropleth map, with percentages of total knowledge gaps calculated per city to show relative burden.

#### Predictive analytics based on generalized linear models (GLM)

To identify factors associated with reporting all three knowledge gaps (basic theory, professional knowledge, and practical skills), a GLM with a binomial family and logit link was used. The dependent variable was a binary indicator of whether a healthcare worker reported all three knowledge gaps (1 = yes, 0 = no). Independent variables included gender, professional seniority, geographic region (seven regions of Henan), and facility type (five levels), all dummy coded with appropriate reference categories. All categorical variables were dummy coded with appropriate reference categories. The logit link function is expressed as:


$$\begin{array}{l} logit\left(p\right)=log\left(p/\left(1--p\right)\right)=\text{β}0+\text{β}1X1+{\text{β}}_{2}X_{2}+...+{\text{β}}_{k}X_{k} \end{array}$$ (1)

Where ‘*p*' represents the probability of reporting all training gaps, and X_1_, X_2_, ..., X_k_ are the predictor variables including demographic and institutional characteristics. The full model specification is:


$$\begin{array}{l} \log\left(\frac{p_{i}}{1-p_{i}}\right)=\beta_{0}+\overset{k}{\underset{j=1}{\sum }}\beta_{j}X_{ij} \end{array}$$ (2)

Where:

*p*_*i*_: Probability that the ith healthcare worker reported “all knowledge gaps”

β_0_: The intercept

β_*j*_: Regression coefficients

*X*_*ij*_: Predictor variables (Individual and institutional characteristics)

Model fit was assessed using the Akaike Information Criterion (AIC) and Bayesian Information Criterion (BIC), with lower values indicating better fit.

## Results

### Characteristics of respondents

A total of 119,051 primary healthcare workers from across all 17 prefectures of Henan province participated in the study. Table 1 presents the demographic characteristics of the respondents. The sample included healthcare workers from various facility types, with Village Health Clinics (*n* = 54,138; 46.5%) and Township Health Centers (*n* = 47,937; 40.27%) representing the majority, followed by Community Health Service Centers (*n* = 15,193; 12.76%) and Health Management Institutions (*n* = 274; 0.23%). The distribution of respondents by professional seniority indicates that nearly half were at the junior level (*n* = 55,151; 46.33%), followed by those at the intermediate level (*n* = 25,407; 21.34%) and a smaller proportion at the associate senior level (*n* = 2,570; 2.16%) and senior level (*n* = 375; 0.31%). Gender analysis revealed a higher representation of female healthcare workers (*n* = 63,877; 53.66%). Geographically, responses covered all regions of Henan province, with Central Henan (*n* = 28,232; 23.71%), and Eastern Henan (*n* = 20,556; 17.27%) accounting for the largest shares.

**Table 1:** Demographic characteristics of primary healthcare workers in Henan province (*N* = 119,050).

**Characteristics**	***n***	**%**
**Gender**		
Female	63,877	53.66
Male	54,934	46.14
Missing	239	0.20
**Facility type**		
Village health clinics	54,138	45.48
Township health centers	47,937	40.27
Community health service centers	15,193	12.76
Health management institutions	274	0.23
Others	1,508	1.27
**Professional seniority**		
Junior level	55,151	46.33
Intermediate level	25,407	21.34
Associate senior level	2,570	2.16
Senior level	375	0.31
Unclassified	35,547	29.86
**Geographic region**		
Northern Henan	16,371	13.75
Central Henan	28,232	23.71
Eastern Henan	20,556	17.27
Western Henan	9,971	8.38
Southern Henan	16,491	13.85
Northwest Henan	1,096	0.92
Northeast Henan	3,487	2.93
South-Central Henan	13,567	11.40
Missing	9,279	7.79

Analysis of reported knowledge gap combinations revealed that a majority of primary healthcare workers reported multiple deficiencies simultaneously (Table 2). The most prevalent pattern was the co-occurrence of all three major gaps, that is, insufficient basic theoretical knowledge, professional knowledge, and practical skills which was reported by 43,849 individuals (40.0%). Singular gaps were also common, with 17,238 participants (15.7%) reporting only a lack of practical skills and 15,504 (14.1%) reporting only a lack of professional knowledge. A further 11,258 respondents (10.3%) reported lacking both professional knowledge and practical skills. Notably, only 2,428 participants (2.2%) indicated no knowledge gaps at all.

**Table 2:** Distribution of knowledge gap combinations among primary healthcare workers in Henan province.

**Gap combination**	**Count**	**Percentage (95% CI)**
Selected all three gaps	43,849	40.0% (39.7–40.3)
Insufficient practical skills only	17,238	15.7% (15.5–15.9)
Insufficient professional knowledge only	15,504	14.1% (13.9–14.4)
Insufficient professional knowledge and insufficient practical skills	11,258	10.3% (10.1–10.5)
Insufficient basic theory only	9,667	8.8% (8.7–8.9)
Insufficient basic theory and insufficient professional knowledge	6,885	6.3% (6.1–6.4)
Insufficient basic theory and insufficient practical skills	2,727	2.5% (2.4–2.6)
No gaps reported	2,428	2.2% (2.1–2.3)

**Figure 2:**
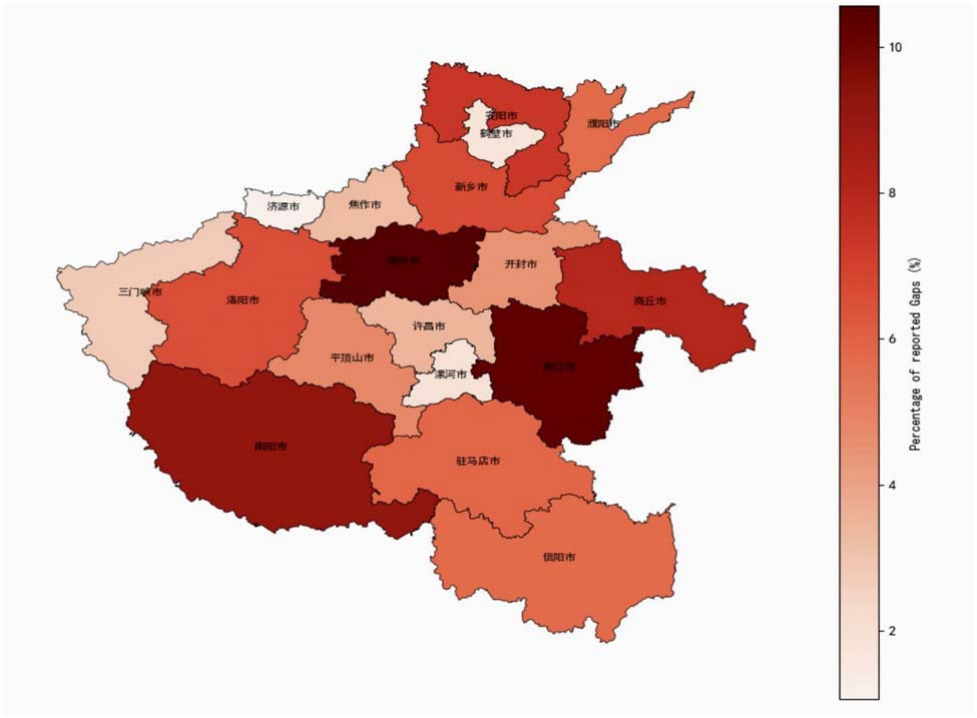
The distribution of reported knowledge gaps across Henan province.

The distribution of reported knowledge gaps revealed variation across Henan province (Figure 2). Cities such as Zhengzhou (郑州市), Nanyang (南阳市), and Zhoukou (周口市) exhibited the highest total number of reported training gaps, collectively accounting for a significant proportion of the provincial burden. In contrast, cities like Sanmenxia (三门峡市) and Luohe (漯河市) reported relatively lower needs. The choropleth map illustrates these differences, with darker shades highlighting areas of concentrated knowledge gaps.

### Extent of reported training needs across facility types: “All Gaps” vs. “No Gaps”

**Figure 3:**
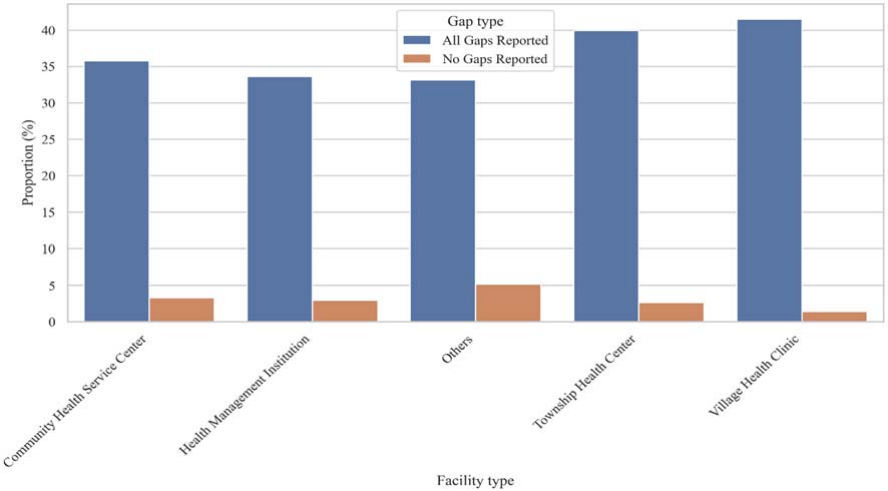
Distribution of self-reported knowledge and skills gaps among primary healthcare workers, by facility type.

After adjusting for the number of respondents per facility type, Village Health Clinics and Township Health Centers had the highest proportions of staff reporting all three core knowledge gaps; basic theory, professional knowledge, and practical skills (over 40%). In comparison, Community Health Service Centers, Health Management Institutions, and Other facilities showed slightly lower rates (33–36%). Reports of having no knowledge gaps were rare across all settings, with the highest in “Other” facilities (5.14%) and the lowest in Village Health Clinics (1.44%). These findings point to a concentrated need for capacity-building in rural and grassroots facilities (Figure 3).

### Sub-group analyses of self-reported knowledge gaps

**Figure 4:**
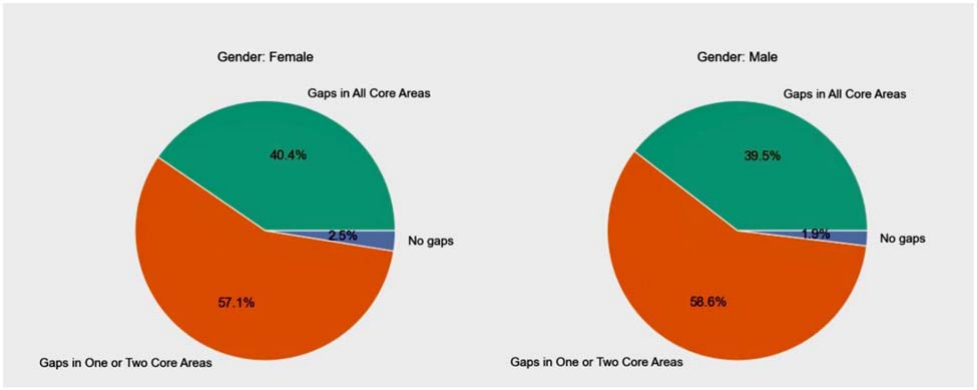
Distribution of self-reported competency gaps among primary healthcare workers by gender.

The distribution of knowledge gaps across gender revealed similar patterns among male and female healthcare workers (Figure 4). A majority of both groups reported gaps in one or two core areas 57.1% among females and 58.6% among males. Approximately, 40.4% of female respondents and 39.5% of male respondents reported gaps in all core areas, including basic theory, professional knowledge, and practical skills. Only a small proportion of each group reported no knowledge or skill gaps, with slightly more females (2.5%) than males (1.9%). Subgroup analysis by seniority revealed that junior-level staff reported the highest prevalence of knowledge gaps in both professional knowledge (70.3%) and basic theory (57.9%), with practical skills gaps also notably high (69.0%). Intermediate and associate senior personnel showed relatively lower but still substantial gaps across all domains, particularly in professional knowledge and practical skills. Interestingly, senior-level staff reported slightly higher gaps in professional knowledge (67.6%) compared to their intermediate and associate senior counterparts, though their practical skills gap was the lowest (59.0%).

### Training preferences by facility type

**Figure 5:**
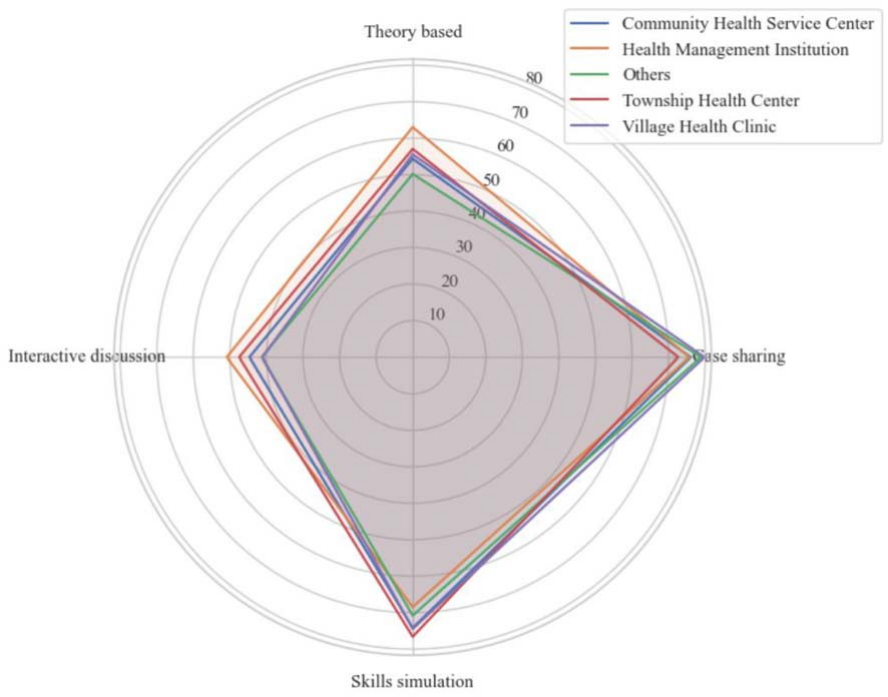
Training modality preferences among primary healthcare workers across facility types in Henan province.

The analysis of training preferences among primary healthcare workers revealed a consistent pattern across facility types (Figure 5). Case sharing emerged as the most preferred training modality, with high selection rates across all facilities, including Village Health Clinics (79.74%), “Other” facilities (78.78%), and Health Management Institutions (75.63%). This was closely followed by simulation-based training, with notable uptake among workers in Township Health Centers (76.61%) and Village Health Clinics (74.47%). Theory-based explanations, while still relevant, were less popular, particularly in “Other” facilities (50.18%) and Community Health Service Centers (54.36%), suggesting a general shift away from didactic formats.

### Mapping knowledge gaps to training preferences

**Figure 6:**
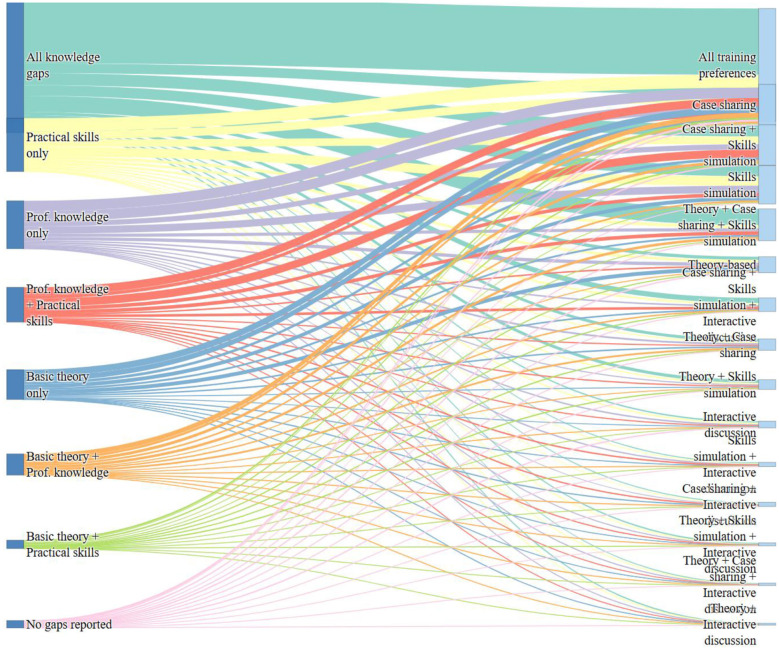
Sankey diagram showing the relationship between self-reported knowledge gaps and preferred training modalities among primary healthcare workers.

The Sankey diagram (Figure 6) illustrates the mapping of the self-reported knowledge gaps and preferred training modalities among PHCWs. Respondents who selected “All Knowledge Gaps” overwhelmingly preferred a combination of theory-based instruction, case sharing, and skills simulation, indicating a demand for multifaceted training approaches. Those reporting deficiencies in professional knowledge or practical skills similarly favored experiential learning formats particularly skills simulation and case sharing. Even among those with specific or limited gaps, for example only in basic theory or practical skills, preferences leaned heavily toward interactive and applied learning methods over traditional theory-based instruction alone.

### Association between training preferences and PHC variables

Chi-square tests were used to assess the associations between participants' demographic characteristics and training preferences (Table 3). The results revealed that gender was significantly associated with all the training preferences. Facility type also showed strong associations across all training modalities, particularly for “Skills Simulation” and “Interactive discussion.” Professional seniority was significantly associated with preferences for theory-based training (χ^2^ = 108.7, *p* < 0.001), skills simulation (χ^2^ = 29.1, *p* < 0.001), and interactive discussion (χ^2^ = 85.4, *p* < 0.001). Junior and intermediate-level staff were more likely to prefer these formats, while case-sharing were consistent across seniority levels. Regional training preferences varied significantly, with central, eastern, and western Henan showing the most differences (all *p* < 0.01), especially in central Henan (e.g., χ^2^ = 55.68, *p* < 0.001 for case sharing). In contrast, Southern, Northwest, Northeast, and South-Central Henan exhibited more consistent preferences across training types.

**Table 3:** Chi-squared test for association between demographic variables and training preferences.

**Variable**	**Training preference**	**χ2**	**df**	***p*-value**
Gender	Theory-based	7.08	1	0.0052^**^
	Case-sharing	46.84	1	
	Skills simulation	200.94	1	
	Interactive discussion	295.66	1	
Seniority	Theory-based	150.76	3	0.001^***^
	Case-sharing	22.32	3	
	Skills simulation	29.37	3	
	Interactive discussion	97.50	3	
Region	Theory-based	72.62	6	0.0097^**^
	Case-sharing	35.67	6	
	Skills simulation	16.89	6	
	Interactive discussion	34.74	6	
Facility type	Theory-based	55.54	3	0.001^***^
	Case-sharing	661.57	3	
	Skills simulation	84.90	3	
	Interactive discussion	388.40	3	

^**^means the result is statistically significant at the 1% level (*p* < 0.01). ^***^means the result is statistically significant at the 0.1% level (*p* < 0.001).

### Clustering analytics and cross-analysis between knowledge gap and training preferences

We applied unsupervised machine learning using K-Means clustering to identify patterns of knowledge gaps as well as training preferences. A 6-cluster solution was found to be optimal (silhouette score = 0.928) for knowledge gap and 6 clusters (silhouette score = 0.743) for training preferences. Table 4 summarizes clustering performance metrics for varying numbers of clusters (K) using participants' knowledge gaps and training preferences. For both dimensions, clustering quality improved consistently as K increased. The six-cluster solution demonstrated the best performance across all indices for both models. Specifically, knowledge gap clusters achieved the highest silhouette score (0.928), highest Calinski-Harabasz index (38,796.44), and lowest Davies-Bouldin index (0.307), indicating excellent compactness and separation. Similarly, the training preference model attained its optimal performance at K = 6, with improved silhouette (0.743), higher Calinski-Harabasz (116,178.51), and lower Davies-Bouldin (0.615), supporting the choice of six clusters for subsequent analyses.

**Table 4:** Clustering evaluation metrics for knowledge gaps and training preference dimensions across different numbers of clusters (K).

**Knowledge gaps clusters**					**Training preference clusters**			
**K**	**Inertia**	**Silhouette**	**Calinski-Harabasz**	**Davies-Bouldin**	**Inertia**	**Silhouette**	**Calinski-Harabasz**	**Davies-Bouldin**
2	210,529.55	0.491	61,476.12	1.246	272,237.10	0.453	66,796.66	1.119
3	118,486.29	0.649	97,167.51	0.909	185,067.53	0.544	74,929.54	1.048
4	71,055.93	0.746	132,392.97	0.678	132,040.61	0.621	84,678.50	0.882
5	37,113.81	0.850	215,149.35	0.483	102,498.66	0.662	89,706.17	0.719
6	17,589.16	0.928	387,496.44	0.307	69,531.40	0.743	116,178.51	0.615

**Figure 7:**
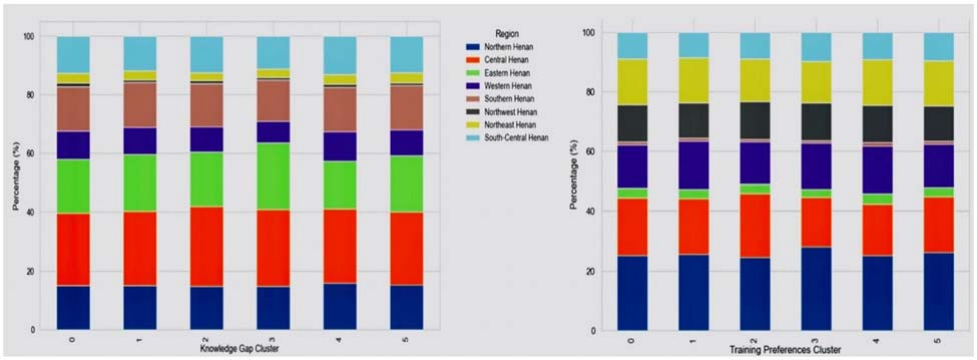
Regional distribution of primary healthcare workers across knowledge gap clusters **(left)** and training preference clusters **(right)**.

The clusters demonstrated distinct profiles of knowledge gaps. The largest cluster exhibited consistent gaps across all three domains basic theory, professional knowledge, and practical skills. Another cluster displayed gaps solely in professional knowledge, while a separate cluster revealed dual deficiencies in theoretical and professional knowledge. One cluster was primarily characterized by a lack of theoretical knowledge, while others were more specialized, with gaps confined to practical skills or a combination of professional and practical skills. Training preferences also varied significantly: the cluster with comprehensive gaps favored all training formats, reflecting a strong demand for blended learning. In contrast, other clusters exhibited more selective preferences, such as a focus on case sharing or skills simulation. Figure 7 illustrates the regional composition of knowledge gaps and training preference clusters. The distribution patterns appear relatively balanced across regions, with no single region overwhelmingly dominating any specific cluster. This may be suggesting that the clustering structures are not driven by regional disparities but rather reflect underlying differences in individual-level knowledge gaps and training preferences. These findings support the generalizability of the cluster profiles across various regions of Henan.

**Figure 8:**
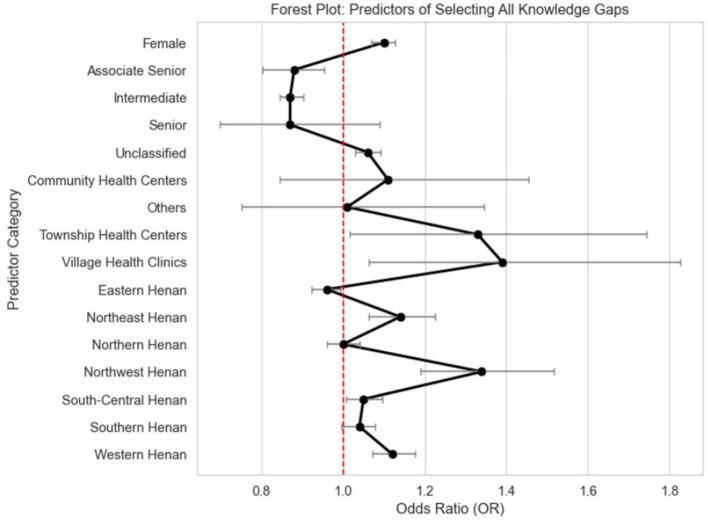
Predictors of selecting all knowledge gaps among primary healthcare workers in Henan province.

Further analyses indicated that the likelihood of reporting all knowledge gaps varied by sex, professional rank, and facility type (Figure 8). Female participants had higher odds compared to male participants (OR = 1.07, 95% CI: 1.07–1.13, *p* < 0.001). Intermediate-level staff had lower odds than juniors (OR = 0.875, 95% CI: 0.803–0.955, *p* = 0.003); seniors showed no significant difference. Staff from Township Health Centers (OR = 1.331, 95% CI: 1.016–1.743, *p* = 0.038) and Village Health Clinics (OR = 1.394, 95% CI: 1.064–1.827, *p* = 0.016) had higher odds of selecting all gaps than Health Management Institution staff.

## Discussion

This study assessed self-perceived knowledge gaps and training preferences among a large population of 119,051 PHC workers in Henan province. The study revealed significant knowledge gaps with nearly half (*n* = 43,849, 40%) of the respondents selecting all three assessed gaps, alongside distinct preferences of learning modalities. This prevalence may be suggesting systemic challenges in both pre-service education and ongoing in-service training programs, aligning with previous reports in China (2), which highlighted widespread quality gaps in Chinese primary healthcare, partly attributable to insufficient competencies of the healthcare provider. The observed patterns of knowledge gaps and training preferences should also be interpreted in light of the broader post-COVID-19 transformation of primary healthcare delivery. The pandemic substantially expanded the scope and intensity of responsibilities borne by primary healthcare workers, while accelerating the adoption of digital tools, remote learning platforms, and task shifting within community-based services. In this context, the strong preference for experiential training modalities such as case sharing and skills simulation may reflect an increased demand for practical, immediately applicable competencies to manage complex and evolving service needs. Rather than representing isolated deficits, the identified knowledge gaps likely signal a workforce adapting to rapidly changing expectations in a health system transitioning from emergency response to long-term recovery and reform. A study by Meng et al. (20) identified a significant “know-do” gap among rural PHC workers in China concerning chronic disease management, emphasizing the disconnect between theoretical knowledge and practical application. The issue of knowledge and skill gaps among PHCWs may not be unique to China as studies from various LMICs have reported similar challenges, often exacerbated by resource limitations, inadequate training infrastructures, and evolving healthcare landscape (38). Research in sub-Saharan Africa has consistently pointed to deficiencies in PHCWs' knowledge regarding common infectious diseases and maternal health protocols (39). Despite significant healthcare investments in Henan, substantial knowledge gaps persist, especially among PHC workers in Village Clinics and Township Health Centers, the primary care providers for much of the rural population, highlighting ongoing challenges in achieving a uniformly competent workforce. This finding aligns with extensive literature documenting the urban-rural divide in healthcare quality and resources in China (40). The observation that junior-level staff reported higher concentrations of knowledge gaps compared to those with intermediate seniority was somewhat expected, reflecting a natural learning curve and accumulation of experience. However, it raises questions about the adequacy of initial training and onboarding processes for new entrants into the PHC workforce. Effective mentorship and structured early-career support could be crucial in mitigating these initial deficits. The finding that female PHC workers had slightly higher odds of reporting all knowledge gaps in the GLM analysis warrants careful interpretation. This could reflect a greater willingness to acknowledge limitations, differences in roles, and responsibilities, or differential access to training opportunities. The spatial variations in knowledge gaps, with cities like Zhengzhou (郑州), Nanyang (南阳), and Zhoukou (周口) showing the highest burden, suggest that resource allocation for training and support may not be evenly distributed or that certain regions face unique challenges, underscoring the importance of geographically targeted interventions.

The pervasive knowledge gaps, especially the discrepancy between theoretical knowledge and practical application, are a clear manifestation of the “Know-Do Gap” (20, 41). The theory posits that possessing knowledge does not automatically translate into its application in practice. Barriers such as lack of confidence, unsupportive work environments, or insufficient practical skills can hinder this translation. The preference for skills simulation directly addresses this gap by providing opportunities for hands-on practice. The overwhelming preference for case sharing and skills simulation over more traditional theory-based lectures and interactive discussions sends a clear message. PHC workers desire learning experiences that are practical, engaging, and directly applicable to their daily work. This aligns strongly with principles of adult learning theory, particularly Knowles' theory of andragogy, which emphasizes that adults are most motivated to learn when they perceive the knowledge as relevant to their life situations and tasks, and when learning is experiential and problem-centered (42). The preference for case-sharing may be suggesting a desire for peer-to-peer learning and the contextualization of knowledge within real-world scenarios. Skills simulation has been shown to be a safe environment to practice and better refine clinical competencies, bridging the “know-do gap” and have been documented in previous studies (43). The variations in training preferences by gender, facility type, seniority, and region further emphasize the need for a tailored approach to training design and delivery. Junior staff or those in rural clinics may have specific preferences, training hence programs should be adapted accordingly, rather than adopting a one-size-fits-all model. In addition, social cognitive theory (44) offers insights into how training can enhance self-efficacy among PHCWs. Observing other successful peers (case sharing), practicing skills (simulation), and receiving constructive feedback can bolster PHCWs' confidence in their ability to perform tasks.

The application of K-Means clustering identified six distinct clusters for both knowledge gaps and training preferences, offering a nuanced understanding beyond aggregate statistics. PHCWs in a cluster characterized by specific skill deficits and a preference for simulation could receive tailored simulation-based training, potentially improving efficiency and impact compared to generic programs. The finding that these cluster structures were not predominantly driven by regional disparities suggests that these individual-level needs and preferences are widespread, reinforcing the need for flexible and personalized training pathways within broader regional strategies.

This study had several notable strengths. The large sample size, covering all 17 prefectures of Henan province, provides robust and generalizable findings for this specific region, which itself may be a significant microcosm of China's broader PHC challenges. The detailed assessment of various knowledge gap combinations and a range of training preferences offers granular insights. The use of advanced statistical methods, including GLM and clustering analysis, adds depth to the interpretation of factors associated with knowledge gaps and the heterogeneity of the PHC workforce.

However, certain limitations must be acknowledged prior to interpretation of these findings. First, the data are based on self-reported knowledge gaps and preferences, which may be subject to social desirability bias or inaccurate self-assessment. Objective measures of knowledge and skills, alongside observed practices, would provide a more complete picture. Second, while Henan province is large and diverse, caution is warranted when generalizing findings to other provinces in China or internationally, given variations in health systems, educational backgrounds, socio-cultural contexts, and other attributes. Third, the questionnaire, while somewhat informed by the Hennessy-Hicks framework, was not a direct application, which might limit direct comparability with studies strictly adhering to that instrument. In addition, although this study was informed by the Hennessy–Hicks Training Needs Analysis framework, the instrument was adapted to reflect the Chinese primary healthcare context, which may limit direct comparability with studies applying the original tool without modification. Finally, the study did not explore the underlying reasons for the reported gaps and training preferences in depth, which could be a valuable avenue for future qualitative research.

### Policy implications for healthy China 2030

The Healthy China 2030 plan highlights a vision for achieving health equity, disease prevention, and universal health coverage across China. The current findings provide workforce-focused evidence that directly supports this national strategy. First, the widespread knowledge gaps among PHCWs, particularly in basic theory, professional knowledge, and practical skills, pose a barrier to achieving the plan's target of increasing national life expectancy to 79 years by 2030. Without a competent frontline workforce, the prevention and management of chronic diseases, which are central to Healthy China 2030, remain compromised. Second, the disproportionate burden of gaps in township and village health facilities underscores persistent urban–rural inequities, echoing Healthy China 2030's emphasis on strengthening grassroots primary care (15). Targeted interventions in rural areas including mobile training teams, regional hubs, and mentorship programs are therefore critical to narrowing these disparities. Third, the preference for experiential learning methods such as case sharing, simulation aligns with the policy's call for innovation in health education and training. Embedding such approaches into continuing medical education can accelerate skill acquisition, enhance the translation of knowledge into practice, and ultimately improve the quality of patient-centered care. Integrating digital health technologies, including mobile platforms and e-learning, can further extend access to training and help fulfill Healthy China 2030's objective of improving health literacy nationwide.

## Conclusion

The study identified significant gaps in basic theory, professional expertise, and practical skills among junior PHC workers, particularly those in township and village clinics, alongside a strong preference for experiential learning methods like case studies and skills simulations. These patterns were observed across a province-wide sample in Henan, reflecting the training needs and workforce characteristics within this specific primary healthcare context. Accordingly, while the findings provide relevant evidence for provincial-level workforce planning, their applicability to other regions should be assessed in relation to local health system structures and training environments. To address these gaps, policymakers should mandate a standardized, competency-based curriculum co-designed with frontline staff and experts, integrating practical skills from the outset and subject to regular review. Dedicated funding is needed for regional training hubs and mobile units to ensure equitable access for rural areas. Continuous professional development (CPD) should include mandatory accredited hours, diverse learning formats such as e-learning, workshops, and peer-led discussions and be clearly linked to career progression. Core training should emphasize experiential methods, while structured supervision and mentorship are essential to support especially new and rural PHC workers.

## Data Availability

The raw data supporting the conclusions of this article will be made available by the authors, without undue reservation.
