# Supplementary material for: Bridging knowledge gaps for healthy China 2030 plan: a province-wide assessment of primary healthcare workers' competencies and training preferences in Henan, China
Source: Front Public Health. 2026 Feb 26;14:1762052. doi: 10.3389/fpubh.2026.1762052 (PMC12979541; doi:10.3389/fpubh.2026.1762052)
Supplement: Supplementary file 1 [file Data_Sheet_1.pdf]

# Research on the Training needs of Primary Healthcare Workers (PHCWs) in Henan, China

**1、Basic Information**

1. Your district and county:
2. Your medical institution:

   ☐ Village clinic

   ☐ Community Health Service Centers\Stations

   ☐ Township Health Centers

   ☐ Health management agencies

   ☐ Other
3. Your professional and technical title:

   ☐ Unrated

   ☐ Elementary

   ☐ Intermediate

   ☐ Deputy Senior

   ☐ Positively advanced
4. Your current main job position:

   ☐ General practitioners, TCM or other clinicians

   ☐ Public health or preventive care

☐ Care

☐ Rehabilitation

☐ Medical technology (pharmacy/traditional Chinese medicine, imaging, testing, etc.).

☐ Administration

☐ Other

5. The number of years you have been working in primary health care is

**2、Survey of the current situation**

6. What gaps do you think need to be filled to improve your service capabilities?

☐ Lack of basic theory

☐ Lack of expertise

☐ Lack of practical skills

☐ Other ________________

7. What you expect from the training is:

| | need | So so | No, you don't |
|---|---|---|---|
| General practice concept service | ○ | ○ | ○ |
| Diagnosis and treatment | ○ | ○ | ○ |

| | | | |
|---|---|---|---|
| of common diseases | | | |
| Basic public hygiene | ○ | ○ | ○ |
| Chronic disease management | ○ | ○ | ○ |
| Rational use of drugs at the grassroots level | ○ | ○ | ○ |
| Professionalism/health regulations | ○ | ○ | ○ |
| Appropriate techniques for traditional Chinese medicine | ○ | ○ | ○ |
| Community Health/Rehabilitation/Nursing | ○ | ○ | ○ |
| Prevention and treatment of sudden infectious diseases | ○ | ○ | ○ |
| Emergency First Aid Techniques | ○ | ○ | ○ |
| Interpretation of ancillary examination results | ○ | ○ | ○ |
| Other (please specify) | ○ | ○ | ○ |

Other (please specify) ________________________________

8. How much time do you think is appropriate to spend on distance

learning each day on average?

☐ Less than 2 hours

☐ 2~4 hours

☐ >4 hours

☐ Other ________________

9. Which of the following learning content appeals to you the most?

☐ cutting-edge content, introducing the latest theoretical knowledge and technical methods

☐ The content is practical, and the knowledge and skills learned can be better applied to practical work

☐ Content specification, summary and refinement of guidelines and consensus

☐ The content is rare, such as the theoretical knowledge of rare diseases, special cases, etc

10. Among the various types of training, you like [Multiple Choice Questions] the most

☐ Theoretical explanation

☐ Case (case) sharing

☐ Skill operation, simulation drills

☐ Interactive discussions, etc

3. Research on **the improvement of grassroots service capacity**

11. Abilities you expect to improve:

| | need | So so | No, you don't |
|---|---|---|---|
| Clinical knowledge and skills | ○ | ○ | ○ |
| TCM knowledge and skills | ○ | ○ | ○ |
| Home care | ○ | ○ | ○ |
| Rehabilitation knowledge and skills | ○ | ○ | ○ |
| Nutrition and health knowledge and skills | ○ | ○ | ○ |
| Health management knowledge and skills | ○ | ○ | ○ |
| Medical Psychology/Counseling | ○ | ○ | ○ |
| Humanities Education/Doctor-Patient Communication Skills | ○ | ○ | ○ |
| Community management capacity | ○ | ○ | ○ |
| Work system and service specifications | ○ | ○ | ○ |
| Other (please specify) | ○ | ○ | ○ |

Other (please specify) ________________________________

12. Clinical theoretical knowledge: (multiple choice, select the ability you think needs to be improved the most)*

□ Basic knowledge of clinical medical theories

□ Basic theoretical knowledge of general practice (general practice concepts, etc.)

□ Relevant community expertise

□ Basic knowledge of TCM

□ Basic pharmacological knowledge and clinical rational drug use knowledge

□ Judgment and interpretation of auxiliary examinations

□ Standardized writing of medical records and other documents

□ Diagnosis and treatment of undifferentiated diseases

□ other

13. Clinical diagnosis and treatment skills: (multiple choices, select the ability you think needs to be improved the most)*

□ Diagnosis and treatment of common and frequently occurring diseases

□ General practice services for chronic diseases

□ Basic First Aid Competencies (Emergency First Aid, Identification, Initial Management, Referral)

□ Evidence-based management of the disease

□ Mastery and application of routine diagnosis and treatment operation techniques

□ Ability to practice general practice

□ History taking techniques

□ Rational use of drugs in the community (including guidance on medication for residents)

□ Appropriate techniques for TCM diagnosis and treatment

□ other

14. Comprehensive medical service capabilities: (multiple choices, select the abilities that you think need to be improved the most)*

□ Initial consultation and referral services

□ Medical Psychology Services (Counseling Related Techniques)

□ Home medical care (home assessment, home bed, special needs house call service, etc.)

□ Rehabilitation medical services (physical, heart, pulmonary, mental, etc.)

□ Clinical thinking and decision-making in general practice

□ Palliative care services

□ Health Assessment Services

□ Fall prevention

□ Health Counseling Methods

□ other

15. Preventive Service Capabilities: (Multiple choices, select the competencies that you think need to be improved the most)*

□ Disease prevention, control and health care

□ Health care for key and special populations (elderly, children, sick, disabled, pregnant, etc.)

□ Health education and health promotion

□ Infectious disease reporting and follow-up management

□ Fever sentinel (pre-inspection and triage, putting on and taking off protective clothing, nucleic acid sampling, fever treatment process, etc.)

□ Community-based diagnostics based on epidemics

□ Assist in handling public health emergencies

□ Health management of key populations/diseases

□ Traditional Chinese medicine treats diseases before they occur

□ Leading Community Health

□ other

16. Improve residents' health literacy ability: (multiple choice, select the ability you think needs to be improved the most)*

□ Nutritional prescriptions

□ Exercise prescription

□ Ways to quit smoking

□ Methods of abstaining from alcohol

□ Mental health education

□ Oral health care

□ Weight management

□ Bone health

□ other

17. Information application ability: (multiple choice, select the ability you think needs to be improved the most)*

□ Establishment, use and management of health records

□ Keep complete medical records

□ Accessing information, tracking, and mastering new technologies

□ Ability to analyze, process and manage information

□ Use of information technology to help diagnose and educate patients (telemedicine, self-media, etc.)

□ Understand the health needs of the community

□ other

18. Health Supervision and Coordination: (Multiple choices, select the abilities that you think need to be improved the most)*

□ Health systems awareness and coordination

□ Evaluation and improvement of health services

□ Occupational safety management (risk avoidance, etc.)

□ other

19. Medical Humanistic Care: (Multiple choices, select the abilities that you think need to be improved the most)*

□ Patient-centered service concept

□ Treat patients with respect

□ Treat patients fairly and equitably

□ Protect patient privacy

□ Humanistic feelings

□ Ability in medical jurisprudence

□ Medical economic decision-making ability

□ Hospice care

□ Bereavement care

□ Tell the bad news

□ other

20. Professional Attitude and Literacy: (Multiple choice, select the ability you think needs to be improved the most)*

□ Love what you do

□ Have the initiative to complete medical work

□ Abide by professional ethics and ethics of conduct

□ Correct professional values

□ Psychological adaptability to medical work

□ Critical thinking skills

□ Medicine-related laws, regulations, and policies

□ other

21. Which of the following would you most like to see your capabilities enhanced? (Select up to 3 items)*

□ Theoretical learning only

□ Skills training only

□ Theoretical learning is the mainstay

□ Skill training is the mainstay

□ Theory meets practice

22. In order to better improve the training effect, do you have any good opinions and suggestions for teaching?

________________________________________________________________
